# HOPE—A UNIQUE MODEL TO PROVIDE ORAL HEALTH SERVICES FOR NURSING HOME RESIDENTS

**DOI:** 10.1093/geroni/igad104.2603

**Published:** 2023-12-21

**Authors:** Sohini Dhar, Rochisha Singh Marwaha, Suman Challa

**Affiliations:** UT Health San Antonio School of Dentistry, San Antonio, Texas, United States; UTHSCSA, San Antonio, Texas, United States; UT Health San Antonio School of Dentistry, San Antonio, Texas, United States

## Abstract

Optimal oral health care is an essential component of primary health care for all individuals but especially among the older adults due to chronic health issues and polypharmacy. Moreover, there are several barriers to oral health care for the older adults that include but are not limited to cost of dental care, lack of perceived need for care, dearth of transportation, and fear for dental care. To address these barriers to access, UT Health San Antonio School of Dentistry launched Holistic Oral Health Program for Elders (HOPE) program that aims to address oral health needs of nursing home residents living within a 65-mile radius of San Antonio, TX. The HOPE team is formed of trained dental and public health professionals who work together as a group to ensure that preventive, palliative, and clinical oral health services are provided to all eligible residents of the nursing home. For successful implementation of the HOPE program, the team efficaciously completed several critical steps while planning and developing the program, such as- establishing a relationship and procuring contracts with several nursing homes, collaborating with social workers and nurses, and following an interprofessional approach to provide oral health care to the nursing home residents. Since its inception, the HOPE program has successfully signed contract agreements with several nursing homes and has addressed the oral health needs of more than 300 nursing home residents. The HOPE program aims to continue serving this vulnerable and underserved population to address their oral health needs.

